# Response to the Letter to the Editor: “Corneal thickness, optic nerve sheath diameter and retinal nerve fiber layer evaluation to assess the risk of cerebral edema in type 1 diabetes in children”

**DOI:** 10.1007/s00592-018-1275-z

**Published:** 2019-01-20

**Authors:** Krzysztof Jeziorny, Arleta Waszczykowska, Dobromiła Baranska, Wojciech Mlynarski, Agnieszka Zmyslowska

**Affiliations:** 1 Department of Pediatrics, Oncology, Hematology and Diabetology, Medical University of Lodz, Lodz, Poland; 2 Department of Ophthalmology and Vision Rehabilitation, Medical University of Lodz, Lodz, Poland; 3 Department of Diagnostic Imaging, Polish Mother’s Memorial Hospital Research Institute, Lodz, Poland

Thank you very much for your interest in our research aimed at evaluating the new and non-invasive methods of imaging the increased risk of cerebral edema in the course of diabetic ketoacidosis (DKA) in children with type 1 diabetes [1]. We realize that there are more advanced methods of corneal thickness measurement, but methods proposed by us can be performed at the bedside which is extremely important for pediatric patients due to the difficult cooperation and often severe clinical condition of patients with DKA. In our study, we have considered the imaging methods which can be used on medical duty in any medical unit and when more advanced neuroimaging methods are not available.

As De Bernardo et al. mentioned, corneal edema with increased central corneal thickness (CCT) observed in patients with a risk of cerebral edema [2] is related to hyperglycemia and state of hydration of the patients. The aim of our study was to measure the CCT using a non-invasive and portable pachymeter which meets the criteria for screening tests. To eliminate the error, the measurements were performed several times and the results were averaged. Considering technical limitations of the device, our measurements were performed by one researcher with the highest care. The investigator paid careful attention that the pachymeter was applied vertically to the central area of cornea [1]. CCT measurement with the pachymeter was also used in our other study performed within the corneal procedures in which the safety of the procedure was determined by the precise measurement of corneal thickness [3].

Moreover, to assess the increased risk of cerebral edema in the course of DKA in children, we proposed the ONSD (optic nerve sheath diameter) measurement in the B-scan technique which has already proved its usefulness in this field [4]. The blooming effect mentioned by De Bernardo et al. is a well-known problem in measuring the small-sized objects by the ultrasound method and almost impossible to avoid. The solution to this problem is performing the examination always using the same and repeatable protocol and verification of the results with computer programs, which was made in our study [1].

The A-scan of ultrasound technique, as the oldest and the simplest method of ultrasound examination is a well-known method of measuring the eye-globe in ophthalmology. The idea of the A technique is to measure the distance from the probe to an object but without its visualization. This technique has been widely used when B-scan imaging had a very low quality. The A-scan technique, so-called “blind” ultrasound method, is perfect for estimating the distance in a long axis and parallel to the ultrasonic wave. It does not seem possible to obtain the width of optic nerve with its sheaths in the parallel direction to the ultrasound wave—it is always transverse or at least oblique. Using the A technique, the position of the measured object cannot be checked and furthermore, if the object is not ideally positioned ahead of the target, the distance cannot be measured reliably. This is especially difficult and unreliable in children and non-cooperating patients in poor general condition. Thus, the B scan—although not perfect and with a small inaccuracy—still seems to be a simple, available, and repeatable method of measuring in the axis not parallel to scanning wave [5].

In conclusion, we are very pleased that the study arouses interest and discussion, but we emphasize that there are still the preliminary data and further studies are needed in larger groups of patients to confirm their effectiveness. However, in our opinion the proposed techniques may serve as potential promising methods for the non-invasive assessment of the increased risk of cerebral edema development in pediatric patients with DKA.
